# A Novel Case of Bifacial Diplegia Variant of Guillain-Barré Syndrome Following Janssen COVID-19 Vaccination

**DOI:** 10.3390/neurolint13030040

**Published:** 2021-08-13

**Authors:** Apoorv Prasad, Gage Hurlburt, Sanjiti Podury, Medha Tandon, Seth Kingree, Shitiz Sriwastava

**Affiliations:** 1 Department of Neurology, Berkeley Medical Center, West Virginia University, Martinsburg, WV 25401, USA; apoorvprasad@gmail.com (A.P.); ghurlburt@osteo.wvsom.edu (G.H.); skingree@soundphysician.com (S.K.); 2 Army College of Medical Sciences, New Delhi 110010, India; sanjiti.1997@gmail.com; 3 Department of Neurology, University of Pittsburgh Medical Center, Pittsburgh, PA 15261, USA; medhatandon22@gmail.com; 4 Department of Neurology, Rockefeller Neuroscience Institute, West Virginia University, Morgantown, WV 26505, USA; 5 West Virginia Clinical and Translational Science Institute, Morgantown, WV 26505, USA; 6 Department of Neurology, Wayne State University, Detroit, MI 48201, USA

**Keywords:** GBS, BFP, COVID-19 vaccine, SARS-CoV-2

## Abstract

Guillain-Barré syndrome (GBS) is an immune-mediated demyelinating disorder which attacks the peripheral nervous system. Antecedent infection or vaccine administration are known to precipitate the onset of this disorder. Its typical presentation leads to a symmetric, rapidly progressive, ascending paresis with associated sensory deficits and impaired reflexes. We present a rare case of a bi-facial diplegia variant of GBS, within four weeks of the COVID-19 vaccination. Due to its chronology, clinical manifestations, and cerebrospinal fluid (CSF) findings, we propose this case to be a rare complication of the COVID-19 vaccination.

A 41-year-old morbidly obese gentleman with no significant medical history presented to the emergency department (ED) with acute onset of urinary retention twelve days after his COVID-19 vaccination (Janssen vaccine). The patient only reported arm soreness, mild fatigue, and chills three days after the vaccination which were self-resolving in a few days. He underwent evaluation in the ED followed by urinary catheterization and was followed up with urology as an outpatient. On day 15 following his COVID-19 vaccination, he returned to the ED with a new onset of left-sided facial droop. He was diagnosed with Bell’s palsy and was discharged with prednisone and valacyclovir. His CT brain showed a colloid cyst and, hence, he was referred to see neurosurgeons as an outpatient (Figure 1).

On day 21 post-vaccination, he presented to the ED with subjective weakness and paresthesias in all extremities. He had difficulty feeding himself and ambulating due to his weakness. He also had new onset of right facial weakness. His urinary retention had resolved. He denied any blurry vision, dysphagia, and headache. Upon physical examination, his vital signs were stable. Neurological examination, including a cranial nerve examination showed bilateral lower motor neuron facial nerve palsy, more prominent on the left compared to right. His deep tendon reflexes were absent bilaterally at the patella and the Achilles with mute plantar responses. Motor strength was 4+/5 (Medical Research Council grade) in all four extremities.

An MRI of the cervical and thoracic spine did not show any abnormal T2 signal changes and his lumbar spine showed no significant degenerative disc disease (Figure 1). He underwent lumbar puncture due to suspicion for Guillain-Barré syndrome (GBS) and showed albumino-cytological dissociation with a CSF cell count of 50/uL (lymphocytes 91%, monocyte 9%, red blood cell counts of 123) and CSF protein of 562 mg/dL. Further corrected cerebrospinal fluid (CSF) protein for the presence of RBC was significantly elevated to 562 mg/dL. Other CSF findings included glucose of 67 mg/dL (serum glucose 109 mg/dL) and negative CSF gram stain, CSF lyme polymerase chain reaction (PCR), and viral PCR testing on CSF, including herpes simplex virus, varicella zoster virus, Epstein-Barr virus, cytomegalovirus (CMV) and Borrelia burgdorferi IgM and IgG. Given the classic clinical picture of GBS in absence of other identifiable etiology for his neurologic disease, additional supportive testing with EMG showed a prolonged distal latency with conduction block and slow conduction velocity in bilateral tibial, peroneal nerve, and absent F waves were supportive for demyelinating GBS. He was started on intravenous immunoglobulin (IVIG) at 2 g/kg over 5 days. Other notable laboratory findings included a white cell count of 15.0 × 10^9^/L, possibly due to recent steroid use and borderline elevated D-dimer. The erythrocyte sedimentation rate, creatine phosphokinase, blood glucose, hepatic and renal function, vitamin B12, folate levels, and thyroid-stimulating hormones were all within normal values, whereas the serum HIV test was negative. Chest X ray was unremarkable with no hilar or mediastinal lymphadenopathy.

Clinically, the patient showed significant improvement after the first dose of 400 mg/kg of IVIG. The patient strength was 5/5 in all muscle groups one day after IVIG treatment. He showed recurrence of his right patellar reflex on day 4 of IVIG treatment and trace right ankle reflex with continued absence of left patellar and ankle reflex. The patient finished 5 days of IVIG treatment with mild side-effects, including nausea and headache. He continued to be significantly ataxic and was intended to be placed in a rehabilitation facility.

Patient followed in outpatient clinic had MRI imaging of brain showed incidental finding of colloid cyst, no facial nerve enhancement, no mass lesion, no abnormal leptomeningeal (Figure 2). Repeat MRI lumbar spine with contrast showed thickening of cauda equina nerve roots suggestive of inflammatory demyelinating neuropathy.

Development of vaccines to protect against SARS-CoV-2 infection has become a public health priority due to the ongoing pandemic. Vaccine development often takes decades; however, due to the pandemic, developing a vaccine to prevent COVID-19 has become a race between humans and the virus [1,2]. Accelerated clinical trials have been conducted to develop a safe and effective vaccine in order to control this pandemic [3]. As of April 2021, three SARS-CoV-2 vaccines have received emergency use authorization by the U.S. FDA. These include Pfizer BioNtech and Moderna vaccines, which use messenger RNA (mRNA) technology, and the Janssen COVID-19 vaccine (Johnson and Johnson), approved on 27th February 2021, which is an adenovirus vector-based vaccine [3,4,5,6,7]. So far, 178 million doses of vaccine have been administered in the U.S. [4].

Guillain-Barré syndrome (GBS) is a polyradiculoneuropathy caused by immune-mediated inflammatory demyelination and axonal damage of peripheral nerve fibers, usually preceded by an infectious process or, in rare instances, certain vaccinations [8,9,10,11]. It is characterized by progressive ascending weakness often accompanied by sensory disturbances (e.g., paresthesia) and diminished or absent deep tendon reflexes. Other clinical features of GBS include cranial nerve deficits (e.g., ophthalmoplegia, facial diplegia, bulbar symptoms) and possible respiratory failure. GBS presents with varied clinical manifestations, thus several subtypes of this disease process exist. Isolated bifacial diplegia with paresthesias (BFP) is a rare variant of GBS, described as acute-onset bifacial weakness associated with distal limb paresthesia and preserved limb weakness in the absence of additional cranial neuropathies [9,12].

Though, most often precipitated following a viral infection, GBS has also been recorded as an adverse reaction to certain vaccines. Various cases of GBS following live attenuated viral vaccines, such as the influenza vaccine, poliovirus, and rabies vaccines, have been reported in the literature [11]. These were most likely facilitated by molecular mimicry. To the best of our knowledge, there are only two reported cases of GBS after receiving the COVID-19 vaccine [13,14]. Recent reports by Márquez et al. described two patients, one of which was in the placebo group, developing GBS within 2 weeks of injection with the Johnson & Johnson COVID-19 vaccine, which incorporates a recombinant, adenovirus serotype 26 vector encoding a full-length and stabilized SARS-CoV-2 spike protein. Waheed et al. reported a case of an 82-year-old female with no significant co-morbidities who developed GBS 2 weeks after her first dose of the Pfizer COVID vaccine. The diagnosis was confirmed on MRI and she was treated with IVIG. This was the first case of GBS following the Pfizer COVID-19 vaccine described in the literature.

We present a case of bifacial diplegia 4 weeks post the Janssen COVID-19 vaccination in a previously healthy 41-year-old male. According to diagnostic criteria proposed by Wakerly et al. [12], our case demonstrates bifacial symmetrical weakness and limb areflexia; absence of limb, neck, or ocular weakness; distal paresthesia at the onset of weakness; and cerebrospinal fluid (CSF) albuminocytological dissociation. We propose this unique case as a rare subtype of GBS in post-COVID-19 vaccination context due to its chronology, clinical manifestations, and CSF findings.

GBS is an immune-mediated polyradiculopathy, which presents with a wide range of neurological symptoms due to the involvement of motor, sensory, and autonomic nerves. Symptoms include symmetric ascending paralysis, absent or reduced deep tendon reflexes, and, in severe cases, respiratory failure [9]. Though the exact pathophysiology is still unknown, immune system stimulation is the most widely accepted theory for the etiology of GBS. Since vaccines are capable of stimulating the immune system, it is biologically plausible that GBS can develop following vaccination due to autoimmunity [11]. The epitope of live or attenuated virus could stimulate antibody or T cell production which can cross react with myelin or axonal glycoproteins of peripheral nerves and lead to immune mediated damage.

While around half of those affected by GBS have a preceding history of an identified infection, whether vaccinations can increase the risk of GBS is less certain. A similar association between vaccination and GBS was previously seen with rabies, H1N1 influenza vaccine, oral polio vaccine, diphtheria, and tetanus toxoid vaccine, Meningococcal immunization [10,11,15,16,17]. Vaccine-related GBS has allegedly been associated with the occurrence of GBS; however, concluded causal associations between vaccines and GBS have been temporal despite individual reports [11]. From the available evidence, there is little support to conclude a causal association between the COVID-19 vaccination and the development of GBS. However, there remains the potential of vaccines to possibly trigger GBS and other autoimmune diseases.

Of the three vaccines approved by the U.S. FDA, Pfizer BioNtech and Moderna vaccines are mRNA vaccines that code for the spike proteins found on the surface of SARS-CoV-2, whereas the Janssen COVID-19 vaccine is a recombinant, replication-incompetent adenovirus vector-based vaccine encoding SARS-CoV-2 spike protein [3,18]. Adenoviruses have been used as vaccine delivery vehicles for foreign gene transfer as their genome is easy to manipulate. Once the vector reaches the cell, it uses the cell’s machinery to display the spike protein on the cell’s surface, which generates a robust immune response [18,19]. These vaccines do not cause infection with COVID-19 nor the virus which is used as the vector. Moreover, adenovirus vector vaccines have been used for decades for infectious diseases caused by the Ebola virus, influenza virus, respiratory syncytial virus (RSV), HIV, mycobacterium tuberculosis, and plasmodium [19].

As with any new vaccine or pharmaceutical product, the reporting of various adverse reactions and post-marketing surveillance of the COVID-19 vaccines is expected to continue for years after the approval for its use in the general population. The side effects predicted for the COVID-19 vaccines range from mild allergic reactions (e.g., itching, rashes, and hives) to more severe reactions, such as the swelling of face, eyes, tongue, weakness, respiratory distress, unconsciousness, and anaphylaxis, which require hospitalization. Neurological symptoms including Bell’s palsy have also been reported post vaccination in select cases [20,21].

There are only two reported cases of GBS noted in the Janssen COVID-19 vaccine trial with one being in the treatment arm and another in the placebo arm [13,14]. However, the benefits of vaccinating against COVID-19 outweigh the risks of developing neurological complications, such as GBS and other autoimmune diseases post-vaccination, as it prevents devastating outcomes of the infection [22,23].

In this case report, we describe a case of BFP, a subtype GBS which was most likely precipitated post-vaccination. The patient was diagnosed based on his clinical presentation, classically abnormal CSF findings of albumino-cytological dissociation, and normal neural axis imaging findings. Electromyography was not performed due to no change in the treatment plan, but it will be considered for the outpatient after four to six weeks for any clinical relapses. The patient responded very well to IVIG treatment with improvement of his symptoms and without any adverse side effects from therapy.

Bilateral facial palsy is a rare presentation and may occur secondary to systemic diseases, such as sarcoidosis, lyme disease, GBS, diabetes, borreliosis, and herpes virus infection. GBS is an important etiology of bilateral facial paralysis as it can have a life-threatening course and requires urgent medical management. Diagnosis of BFP is made based on history and clinical examination; however, other major differentials of facial palsy, such as lyme disease and sarcoidosis, must be ruled out. Patients with GBS are treated with IVIg and plasma exchange. In the case of a BFP variant, additional eye care with night patching and eye lubricants must be provided [24,25].

On 13 April 2021, the FDA also paused the administration of the Janssen COVID-19 vaccine due to concerns for blood clots in six women, including one death from cerebral venous sinus thrombosis. By 12 April, 6.8 million doses of the Janssen COVID-19 vaccine had been administered [26]. Hence, it is highly pertinent for healthcare providers to be extremely vigilant about other possible complications from the Janssen COVID-19 vaccine, especially GBS as early diagnosis and treatment can change the course of this rare disorder.

In this case report, we describe the first reported case of GBS following the COVID-19 vaccination outside of the Janssen COVID-19 vaccine clinical trial and with favorable outcome after IVIG treatment. Adverse events related to the use of new vaccines need to be reported. Given the large number of doses administered and the current situation of the pandemic, we could argue that the benefits of vaccinating the population far outweigh the risk of having an adverse reaction. However, we believe that diligent reporting of these rare adverse events would allow physicians to recognize and treat this rare condition earlier.

## Figures and Tables

**Figure 1:**
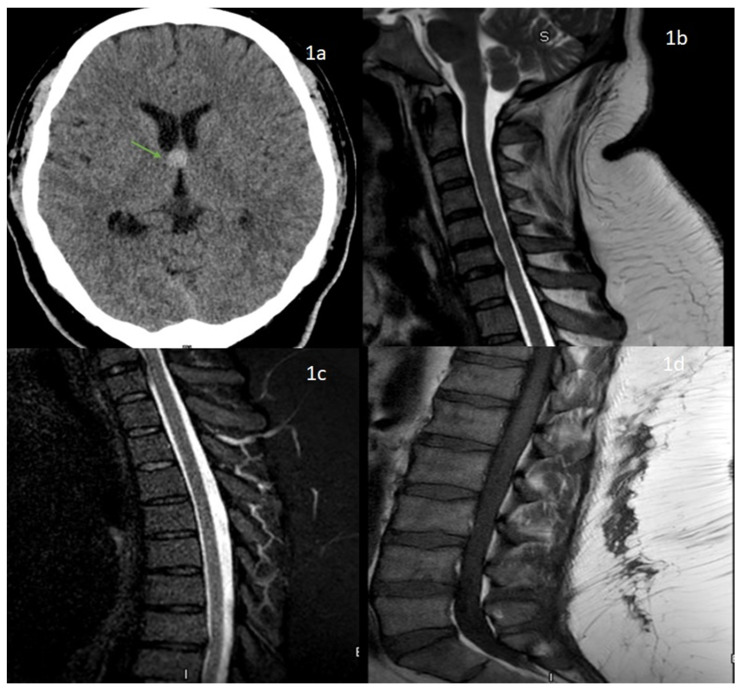
(**1a**) CT head showing colloid cyst at the level of foramen of monroe (green arrow). (**1b**,**1c**) MRI cervical and thoracic spine sagittal images showing no abnormal intramedullary cord signal changes. MRI lumbar spine (**1d**), T1 weighted sagittal image showing no degenerative disc disease.

**Figure 2:**
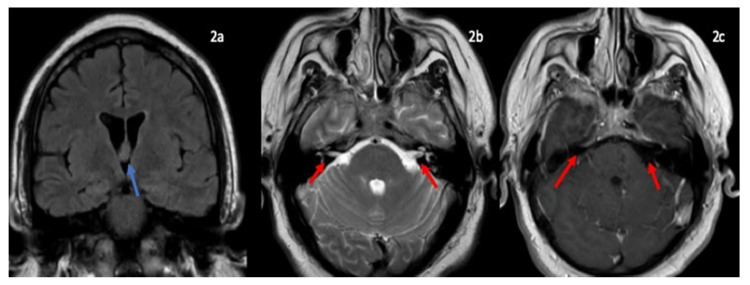
MRI brain coronal FLAIR (**2a**) showing colloid cyst in the foramen of monro (blue arrow). Axial T2-weighted (**2b**) axial T1 post contrast images (**2c**) showing cranial nerve VII and VIII nerve complex (**2b**) and no abnormal enhancment (**2c**) (red arrow).

## Data Availability

Not applicable.

## Abbreviations

SARS-CoV-2	Severe Acute Respiratory Distress Syndrome coronavirus 2
COVID-19	Coronavirus infectious disease-2019
nCov	Novel Coronavirus
AIDP	Acute inflammatory demyelinating polyneuropathy
AMSAN	Acute motor-sensory axonal neuropathy
AMAN	Acute motor axonal neuropathy
BFP	Bifacial weakness with paresthesias
MFS	Miller-Fisher syndrome
GBS	Guillain-Barré Syndrome
IVIG	Intravenous immunoglobulin
EMG	Electromyography
CSF	Cerebrospinal fluid
HIV	Human immunodeficiency virus
WHO	World Health Organization
MRC	Medical Research Council Scale for Muscle Strength
